# Lymph Node Metastasis Around the Common Hepatic Artery Is Associated With Dismal Prognosis in Patients Undergoing Resection of Extrahepatic Cholangiocarcinoma

**DOI:** 10.1002/jhbp.12194

**Published:** 2025-07-29

**Authors:** Sho Kiritani, Yoshikuni Kawaguchi, Yusuke Kazami, Kyoji Ito, Yujiro Nishioka, Yuichiro Mihara, Akihiko Ichida, Takeshi Takamoto, Nobuhisa Akamatsu, Kiyoshi Hasegawa

**Affiliations:** ^1^ Hepato‐Biliary‐Pancreatic Surgery Division, Department of Surgery Graduate School of Medicine, The University of Tokyo Tokyo Japan

**Keywords:** common hepatic artery, extrahepatic cholangiocarcinoma, long‐term survival, lymph node, short‐to‐long axis ratio

## Abstract

**Background:**

Lymph node (LN) metastasis in extrahepatic cholangiocarcinoma (eCCA) is associated with poor prognosis, but the impact of specific metastatic sites is unclear. This study investigated the clinical significance of LN metastasis around the common hepatic artery (N [CHA]) in eCCA.

**Methods:**

A total of 291 patients who underwent curative resection for eCCA between 2002 and 2022 were retrospectively reviewed. Patients were classified as N1 (CHA), N1 (other, regional LN metastasis without CHA), or N0. Clinical characteristics and long‐term outcomes were compared. The short‐to‐long axis ratio (SLR) of CHA nodes on preoperative CT was evaluated for diagnostic value.

**Results:**

Of 291 patients, 164 had perihilar and 127 had distal cholangiocarcinoma. The N1 (CHA), N1 (other), and N0 groups included 33, 103, and 155 patients, respectively. Five‐year cancer‐specific survival (CSS) rates were 6.9% (N1 [CHA]), 24.7% (N1 [other]), and 60.3% (N0). N1 (CHA) and N1 (other) had CSS hazard ratios of 3.34 and 1.86, respectively (*p* < 0.01). The area under the receiver operating characteristics curve for SLR in predicting N1 (CHA) was 0.779.

**Conclusions:**

N1 (CHA) is a strong negative prognostic factor in eCCA. CHA node status may serve as a useful imaging‐based marker of biological resectability.

## Graphical abstract

Kiritani and colleagues identified lymph node metastasis around the common hepatic artery as a powerful independent predictor of poor survival in extrahepatic cholangiocarcinoma. Readily detectable on imaging and accessible during surgery, common hepatic artery node status provides a practical biomarker to refine biological resectability and guide multimodal treatment strategies.

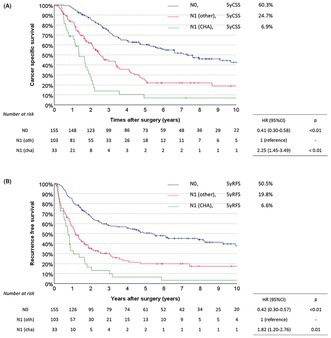

## 1 Introduction

The global incidence of cholangiocarcinoma has increased since the 21st century [1]. Extrahepatic cholangiocarcinoma (eCCA, including perihilar cholangiocarcinoma [PhCC] and distal cholangiocarcinoma [DCC]), which accounts for approximately 80% of all cholangiocarcinomas, is a highly aggressive malignancy that is usually diagnosed at an advanced stage and poses a significant risk of curative resection because of its complex anatomical location. Consequently, the reported 5‐year survival rate remains low, ranging from 2% to 30% [2].

Surgical resection is the only potentially curative treatment for nonmetastatic eCCA. However, recent advances in chemotherapy have caused gradual improvements in outcomes through multimodal treatment strategies [3]. To further enhance treatment options, it is essential to establish criteria for resectability that incorporate anatomical and biological factors, similar to the approach used in pancreatic cancer [4].

Lymph node metastasis is a well‐established poor prognostic factor in eCCA and a significant biological determinant of resectability [5, 6]. The latest Union for International Cancer Control (UICC) staging system classifies nodal status based on the number of metastatic lymph nodes [7]. However, accurately diagnosing lymph node metastasis preoperatively remains a significant clinical challenge [8].

Among regional nodes, metastasis to lymph nodes along the common hepatic artery (N [CHA]) occurs frequently [9]. While these are regional lymph nodes, they are positioned at a site relatively remote from the primary tumor. Additionally, they have a relatively consistent physiological size, making them readily identifiable on preoperative imaging. Intraoperative frozen section analysis enables early assessment during the procedure, providing a timely biological checkpoint.

In light of the aforementioned characteristics of this lymph node, we evaluated the prognostic impact of N (CHA) metastasis in eCCA, and the diagnostic accuracy for detecting N (CHA) metastasis using preoperatively assessable parameters.

## 2 Methods

### 2.1 Study Populations

This retrospective cohort study was approved by the Institutional Review Board of The University of Tokyo Hospital (approval number: 2158‐(11)). Consecutive clinical data were retrieved from a prospectively maintained database of patients with histologically proven eCCA, including PhCC and DCC, who underwent curative resection (defined as R0 or R1 resection) at our institution between January 2002 and December 2022. Patients with distant or lymph node metastases beyond the regional lymph nodes were excluded from the analysis. Based on pathological findings, the patients were classified into three groups: the N1 (CHA) group, comprising those with metastasis to lymph nodes around the common hepatic artery (CHA); the N1 (other) group, comprising those with metastasis to other regional lymph nodes that do not involve the CHA node; and the N0 group, comprising patients without lymph node metastasis.

### 2.2 Treatment Strategy

For preoperative examinations, dynamic contrast‐enhanced computed tomography (dCECT) and endoscopic retrograde cholangiopancreatography‐guided mapping biopsies were performed to assess tumor progression. The dCECT protocol at our hospital was as follows. The contrast medium was injected through the antecubital peripheral vein for 30 s. The concentration and volume of the contrast medium were determined based on body weight: for patients weighing < 50 kg, 300 mg I/mL at a dose of body weight × 2 mL; for those weighing 50–60 kg, 350 mg I/mL at a fixed volume of 100 mL; for those weighing > 60 kg, 370 mg I/mL at a fixed volume of 100 mL. Image acquisition was performed at 25 s (arterial phase), 40 s (portal phase), and 90 s (delayed phase) after contrast injection. The reconstruction parameters were as follows: field of view, 35–40 cm (adjusted according to body size); slice thickness/interval, 1/1 mm. Depending on the case, intraductal ultrasonography and peroral cholangioscopy were performed to determine the surgical approach that would allow R0 resection.

Preoperative treatment was not performed. Hepatic resection with extrahepatic bile duct resection (Hr‐BDR) was initially considered for PhCC, and pancreaticoduodenectomy (PD) was selected for DCC. However, hepatopancreatoduodenectomy (HPD) was actively performed for widespread cholangiocarcinoma [10]. Hepatectomy was primarily planned as a major hepatectomy whenever feasible. Portal vein embolization was considered when the future liver remnant to total liver volume ratio was < 0.4 in those with normal liver function (ICGR15 < 10%) or < 0.5 in those with evidence of liver injury (ICGR15 ≥ 10%). In cases of jaundice, preoperative biliary drainage was performed, and since 2010, endoscopic drainage alone has been adopted as the standard practice [11]. The criteria for resectability included preoperative imaging indicating anatomically feasible R0 resection; no lymph node metastasis beyond regional nodes; absence of distant metastases. In patients requiring concomitant liver resection, preserved remnant liver function was also required for resection eligibility.

During surgery, the absence of unresectable factors, such as liver metastases, para‐aortic lymph node metastases, and peritoneal dissemination, was first confirmed. In the case of PD, regional lymph node dissection was performed, and the bile duct margin was examined intraoperatively using frozen section analysis to confirm negative results. If positive, an additional bile duct resection or HPD was performed. Regional lymph node dissection was performed for Hr‐BDR, and the hepatic and duodenal bile duct margins were examined intraoperatively for negative results. If positive, an additional resection or HPD was performed. Regional lymph nodes were defined as follows: (i) For PhCC: Station 12 (lymph nodes along the hepatoduodenal ligament), Station 8 (lymph nodes around the CHA), and Station 13a (posterior superior pancreaticoduodenal lymph nodes); (ii) For DCC: Stations 12, 8, and 13 (posterior pancreaticoduodenal lymph nodes), Station 17 (anterior pancreaticoduodenal lymph nodes), and Station 14 (lymph nodes around the superior mesenteric artery) [12]. Although adjuvant chemotherapy was not routinely administered, gemcitabine‐based chemotherapy was considered in patients with R1 resection or pathological lymph node metastasis [13].

Postoperatively, tumor markers (carbohydrate 19‐9 [CA19‐9] and carcinoembryonic antigen) were measured every 3 months. The dCECT was performed at 6‐month intervals to assess recurrence. If recurrence was suspected, additional examinations, such as magnetic resonance imaging, positron emission tomography, and histological assessment, were performed to confirm the diagnosis.

### 2.3 Assessment of N (CHA) Parameter

The diagnostic accuracy of N1 (CHA) was evaluated using the following parameters.

**FIGURE 1:**
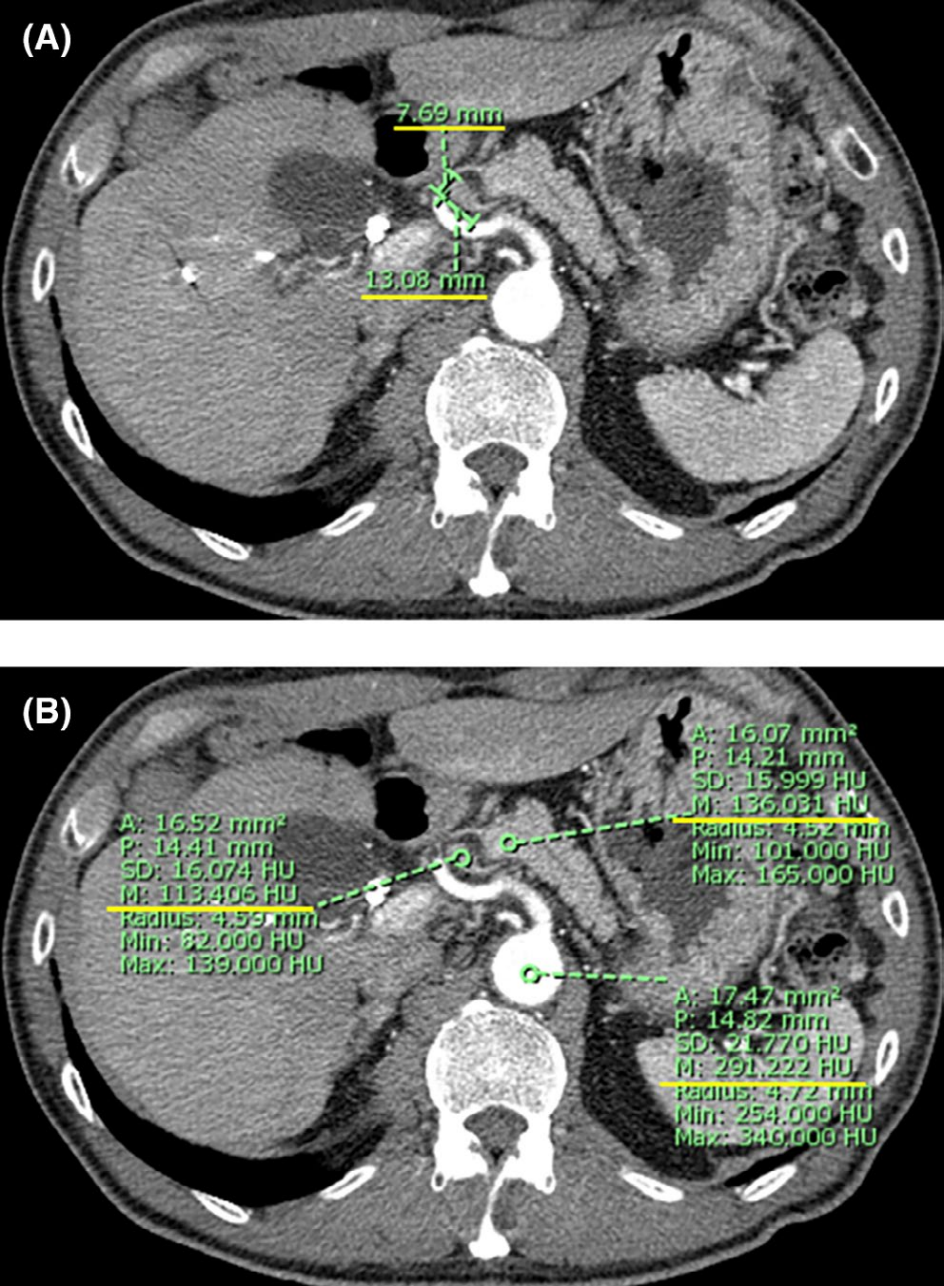
Measurement of N (CHA) parameters on dynamic contrast‐enhanced computed tomography (dCECT). (A) Short‐to‐long axis ratio (SLR): N (CHA) refers to lymph nodes located along the common hepatic artery (CHA). The axial slice of the arterial‐phase dCECT showing the maximum long‐axis diameter of the node; the short‐axis diameter is measured perpendicular to the long axis. SLR was defined as the short‐axis diameter divided by the long‐axis diameter. In this example, 7.69/13.08 = 0.59. This parameter represents the roundness of the lymph node. (B) Hounsfield unit (HU) ratios—HU (LN‐aorta) and HU (LN/pancreas): A circular region of interest (ROI) was positioned around the N (CHA) node to calculate its mean HU value. The mean HU values of the aorta and pancreatic parenchyma are measured on the same slice, and the ratios HU (LN/aorta) and HU (LN/pancreas) were calculated. In this example, HU (LN/aorta) = 113.406/291.222 = 0.39, and HU (LN/pancreas) = 113.406/136.031 = 0.83. These parameters reflect the contrast enhancement of the lymph node.

(i) Short‐to‐long axis ratio (SLR; Figure 1A):

Lymph nodes adjacent to the CHA, appearing elliptical on the arterial phase of dCECT in the axial view, were identified as N (CHA). The longest diameter on the axial section was measured, along with the shortest diameter perpendicular to the longest diameter on the same image. The SLR was defined as the maximum short diameter divided by the maximum long diameter. This value indicates how close the lymph node is to a spherical shape.

(ii, iii) Hounsfield Unit (HU) Values (Figure 1B):

A circular region of interest was placed within N (CHA) on the same image, and the mean HU value was calculated using SYNAPSE VINCENT software (Fujifilm Medical Co., Tokyo, Japan). The mean HU values of the pancreatic parenchyma and aorta were measured on the same image, and the HU (LN/aorta) and HU (LN/pancreas) ratios were determined.

(iv) Serum CA19‐9 Level:

The fourth parameter was the preoperative serum level of CA19‐9, a commonly used tumor marker in eCCA.

### 2.4 Endpoints

Postoperative long‐term survival was evaluated using cancer‐specific survival (CSS) and recurrence‐free survival (RFS). CSS was calculated from the date of surgery to cancer‐related death, with censoring at the last follow‐up date for patients who remained alive or had died from other causes. RFS was calculated from the date of surgery to recurrence identification through imaging or histological examination, or death, with censoring at the last follow‐up date for patients who were alive without recurrence. Postoperative short‐term outcomes were evaluated based on the operative time, blood loss, postoperative complications, and postoperative hospital stay. Postoperative complications were evaluated following the Clavien–Dindo classification [14].

### 2.5 Statistical Analysis

Continuous and categorical variables are expressed as medians, ranges, and percentage frequencies. The Kruskal–Wallis or chi‐square test was used to compare the three study groups. Bonferroni correction was used for multiple comparisons in the Kruskal–Wallis test, and adjusted standardized residuals (ASR) were calculated for the chi‐square test when *p* < 0.05. A receiver operating characteristic (ROC) curve was drawn with N1 (CHA) as the dependent variable and SLR, HU (LN/aorta), HU (LN/pancreas), and CA19‐9 values as the independent variables. The area under the curve (AUC) was calculated. The Kaplan–Meier method was used to estimate the survival rate between the three groups, which was compared using Cox proportional hazards model analysis. Variables with *p* < 0.05 were selected for multivariable analysis using a Cox proportional hazards model with stepwise backward elimination. *p* < 0.05 were considered statistically significant. When the ASRs were ≥ ±2.0, the difference was considered significant. Statistical analyses were conducted using the IBM SPSS Statistics software (version 29.0; IBM Japan Ltd., Tokyo, Japan).

## 3 Results

### 3.1 Patient Flowchart

**FIGURE 2:**
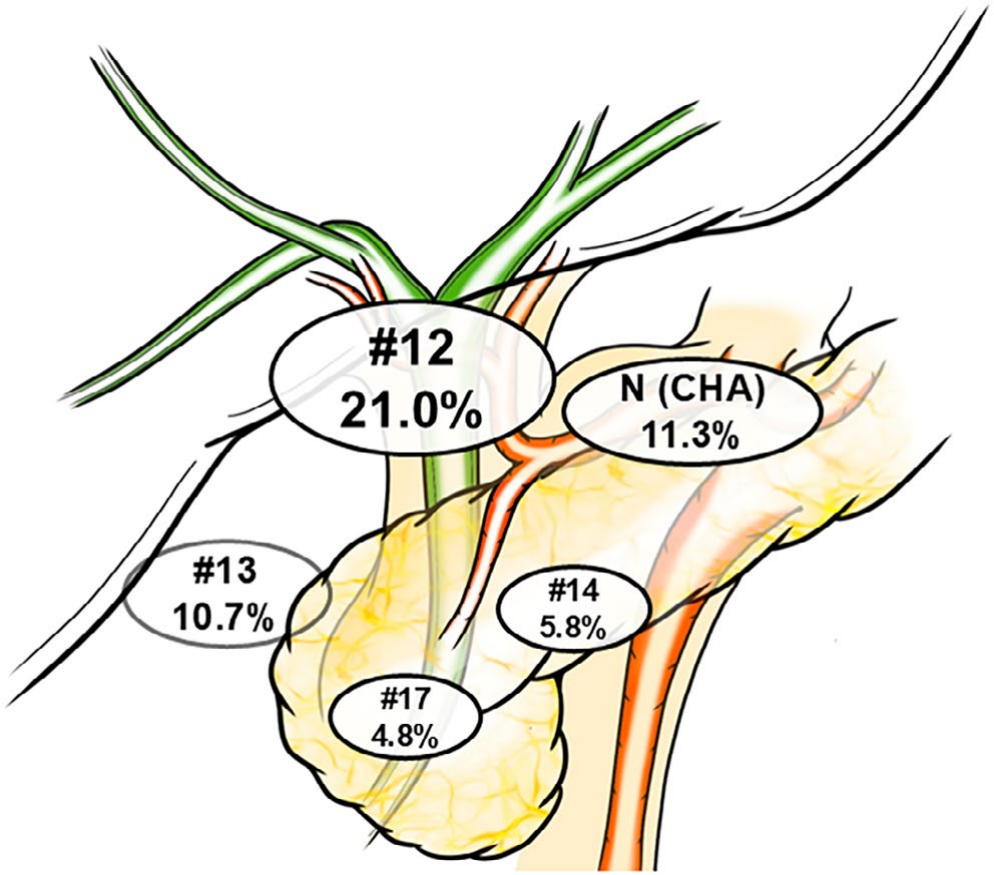
Detailed distribution of metastatic rates based on lymph node station. Cases with positive metastasis to #12, #13, #14, or #17 nodes but negative for N (CHA) were classified as N (other).

During the study period, 291 patients with eCCA underwent curative resection. Of these, 164 and 127 patients had PhCC and DCC, respectively. In the PhCC group, major hepatectomy was performed in 97.6% of cases. Right hepatectomy was performed in 87 cases (53.0%), right trisectionectomy in 2 cases (1.2%), left hepatectomy in 52 cases (31.7%), and left trisectionectomy in 19 cases (11.6%). In the PhCC group, portal vein resection and reconstruction were performed in 29 cases (17.7%), while in the DCC group, this was done in 4 cases (3.1%). Arterial resection was performed in 13 cases (7.9%) in the PhCC group and in 1 case (0.8%) in the DCC group. Among all patients, 33, 103, and 155 were in the N1 (CHA), N (other), and N0 groups, respectively. The breakdown of the N1 (other) group is as follows: 61 patients at Station 12, 31 at Station 13, 14 at Station 17, and 2 at Station 14 (including overlaps). Additionally, 18 patients had metastases spanning two or more regions (Figure 2).

### 3.2 Backgrounds, Perioperative Information, and Pathological Findings

A comparison of the background characteristics and perioperative information of the three groups is presented in Table 1. Jaundice was significantly less frequent in the N0 group (ASR −3.3); however, no significant difference was observed in total bilirubin or CA19‐9 levels. Similarly, surgical procedures showed no significant differences among the groups. The operative time was significantly longer in the N1 (CHA) group than in the N0 and N1 (other) groups (*p* = 0.03 and < 0.01). Additionally, a significant difference was observed in the rate of adjuvant therapy administration (ASR: N0 −2.6, N1 (CHA) 2.3).

**TABLE 1:** Backgrounds and perioperative information between N0, N1 (other), and N1 (CHA) groups.

Variables	N0	N1 (other)	N1 (CHA)	*p*	Adjusted standardized residuals/Multiple comparison
	*n* = 155	*n* = 103	*n* = 33		
Age, years	71 (31–86)	70 (23–84)	69 (36–80)	0.24	
Sex, male, *n* (%)	112 (72.3)	76 (73.8)	21 (63.6)	0.52	
Tumor location, Bp/Bd	90/65	53/50	21/12	0.39	
Jaundice, yes, *n* (%)	113 (72.9)	90 (87.4)	30 (90.9)	< 0.01	N0 −3.3
Preoperative treatment, yes, *n* (%)	1 (0.6)	0 (0.0)	1 (3.0)	0.19	
Total‐bilirubin, mg/dL	1.5 (0.3–29.7)	2.7 (0.5–27.0)	2.9 (0.5/27.5)	0.03	n.s. by multiple comparison
CA19‐9, U/mL	34 (2–3335)	56 (1–34 880)	93 (1–11 950)	0.01	n.s. by multiple comparison
Surgical procedure, PD/Hx/HPD	61/71/23	41/36/26	11/12/10	0.12	
Operative time, min	601 (259–1227)	615 (356–1105)	714 (430–1000)	< 0.01	N0–N1 (CHA) *p* = 0.03, N1 (other)–N1 (CHA) *p* < 0.01
Blood loss, mL	830 (180–8030)	863 (80–4670)	865 (110–2495)	0.96	
Clavien‐Dindo grade, ≥ 3a, *n* (%)	50 (32.3)	38 (36.9)	8 (24.2)	0.39	
Postoperative hospital stays, days	27 (5–106)	27 (10–186)	29 (11–53)	0.84	
Adjuvant treatment, *n* (%)	20 (12.9)	23 (22.3)	11 (33.3)	0.01	N0 −2.6, N1 (CHA) 2.3

*Note:* Data are presented as medians and ranges or numbers and percentages. The analysis was performed using the chi‐square or Kruskal–Wallis test, as appropriate. Bonferroni correction was used for multiple comparisons in the Kruskal–Wallis test, and adjusted standardized residuals were calculated for the chi‐square test when *p* < 0.05.

Abbreviations: CA19‐9, carbohydrate antigen 19‐9; CHA, common hepatic artery; HPD, hepatopancreaticoduodenectomy; Hx, hepatic and extrabile duct resection; PD, pancreaticoduodenectomy.

The pathological findings of the three groups are summarized in Table 2. The T‐factor, based on the 8th edition of the UICC, showed a significant difference among the three groups [7]. Furthermore, tumor differentiation showed variability among the groups. The median number of dissected lymph nodes was 12 in the N0 group, 17 in the N1 (other) group, and 15 in the N1 (CHA) group. Furthermore, when divided into PhCC and DCC, the number of dissected lymph nodes in PhCC was 9 in the N0 group, 14 in the N1 (other) group, and 14 in the N1 (CHA) group. In DCC, it was 18 in the N0 group, 20 in the N1 (other) group, and 20 in the N1 (CHA) group. Significant differences were observed among the three groups regarding microscopic lymphatic, venous, and perineural invasion. Similarly, a significant difference was noted in curative resection status, with the adjusted residuals indicating that the N1 (CHA) group had a significantly lower R0 resection rate (−2.8).

**TABLE 2:** Pathological findings among N0, N1 (other), and N1 (CHA) groups.

Variables	N0	N1 (other)	N1 (CHA)	*p*	Adjusted standardized residuals/Multiple comparison
	*n* = 155	*n* = 103	*n* = 33		
T‐factor^a^					
T1, *n* (%)	31 (20.0)	5 (4.9)	1 (3.0)	< 0.01	N0 4.0, N1 (other) −3.0
T2, *n* (%)	76 (49.0)	41 (39.8)	11 (33.3)		
T3, *n* (%)	48 (31.0)	54 (52.4)	20 (60.6)		N0 −4.0, N1 (other) 2.7, N1 (CHA) 2.3
T4, *n* (%)	0 (0.0)	3 (2.9)	1 (3.0)		N0 −2.1
Tumor differentiation					
Papillary, *n* (%)	1 (0.6)	1 (1.0)	1 (3.0)	< 0.01	
Well, *n* (%)	84 (54.2)	40 (38.8)	10 (30.3)		N0 3.0
Mod, *n* (%)	57 (36.8)	52 (50.5)	12 (36.4)		N1 (other) 2.3
Por, *n* (%)	13 (8.4)	10 (9.7)	10 (30.3)		N1 (CHA) 3.6
Microscopic lymphatic invasion, *n* (%)	51 (32.9)	73 (71.6)	26 (78.8)	< 0.01	N0 −6.9, N1 (other) 5.0, N1 (CHA) 3.3
Microscopic venous invasion, *n* (%)	100 (64.5)	96 (94.1)	32 (97.0)	< 0.01	N0 −6.3, N1 (other) 4.7, N1 (CHA) 2.7
Microscopic perineural invasion, *n* (%)	108 (69.7)	99 (97.1)	31 (93.9)	< 0.01	N0 −5.9, N1 (other) 4.9
Curability, R0, *n* (%)	108 (69.7)	66 (64.1)	14 (42.4)	0.01	N1 (CHA) −2.8

*Note:* Data are presented as medians and ranges or numbers and percentages. The analysis was performed using the chi‐square or Kruskal–Wallis test, as appropriate. Bonferroni correction was used for multiple comparisons in the Kruskal–Wallis test, and adjusted standardized residuals were calculated for the chi‐square test when *p* < 0.05.

Abbreviation: CHA, common hepatic artery.

^a^ The T‐factor was determined based on the Union for International Cancer Control (UICC) 8th edition.

### 3.3 Long‐Term Outcomes

**FIGURE 3:**
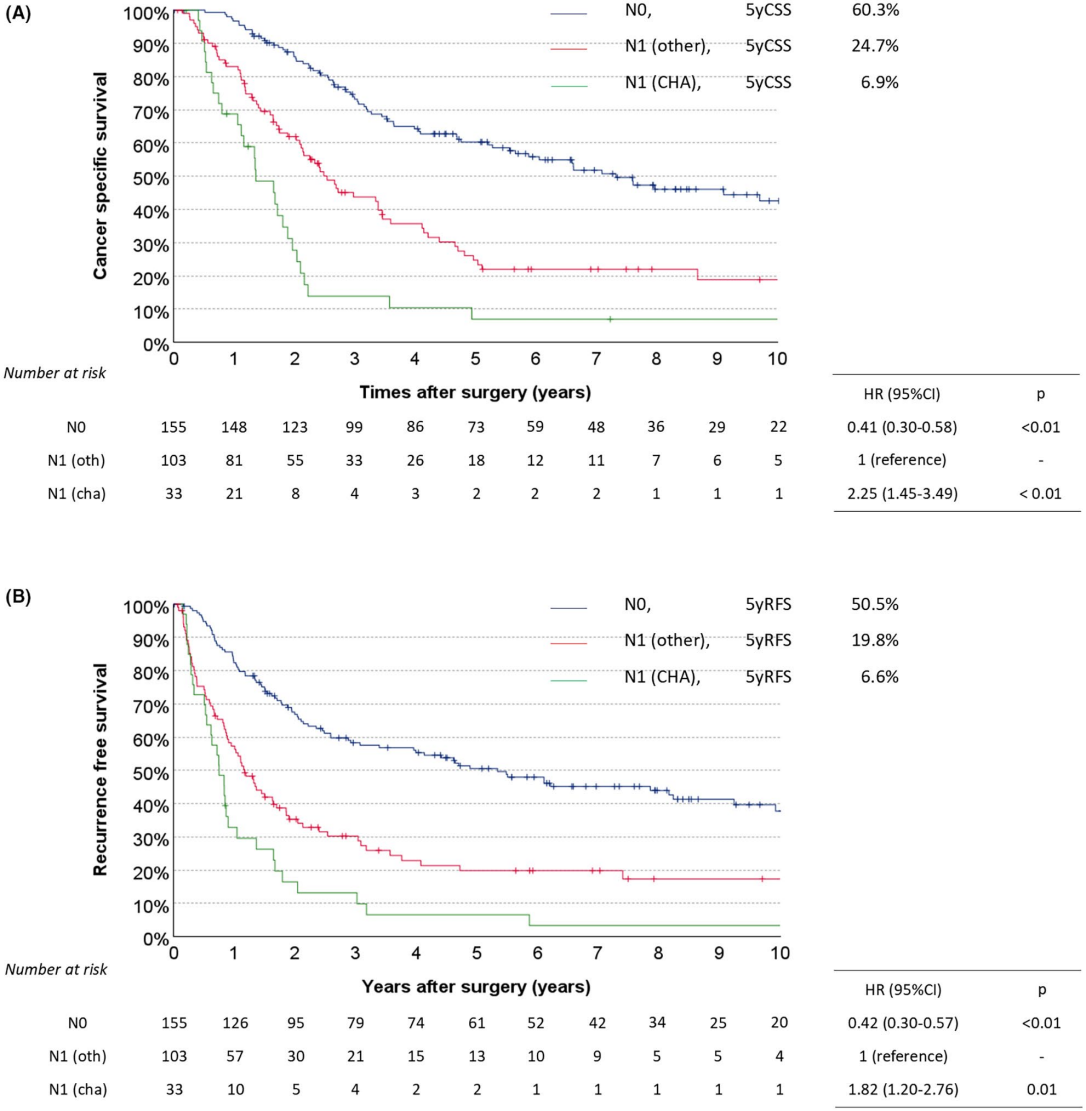
(A) A comparison of cancer‐specific survival curves among the three groups. The univariable hazard ratios (HRs) relative to the N0 are annotated. (B) Comparison of recurrence‐free survival curves among the three groups. The univariable HRs relative to the N0 are annotated.

The median follow‐up period was 5.9 years (range, 0.0–17.4). Figure 3A illustrates the Kaplan–Meier curve used for comparing CSS among the three groups. The 5‐year CSS rates for the N1 (CHA), N1 (other), and N0 groups were 6.9%, 24.7%, and 60.3%, respectively. The hazard ratio (HR) for N1 (CHA), using N1 (other) as the reference, was 2.25 (*p* < 0.01), while the HR for N0 was 0.41 (*p* < 0.01). Figure 3B shows the Kaplan–Meier curve used for comparing RFS. The 5‐year RFS rates for N1 (CHA), N1 (other), and N0 groups were 6.6%, 19.8%, and 50.5%, respectively. The HRs for N1 (CHA), using N1 (other) as the reference, were 1.82 (*p* = 0.01), while the HR for N0 was 0.42 (*p* < 0.01).

Furthermore, the CSS and RFS curves for PhCC are shown in Figure S2A, and those for DCC are shown in Figure S2B. In PhCC, the hazard ratios (HRs) for CSS relative to N1 (other) were 2.17 for N1 (CHA) (*p* = 0.01) and 0.47 for N0 (*p* < 0.01), and the HRs for RFS were 1.71 for N1 (CHA) (*p* = 0.05) and 0.46 for N0 (*p* < 0.01). In DCC, the HRs for CSS relative to N1 (other) were 2.23 for N1 (CHA) (*p* = 0.02) and 0.34 for N0 (*p* < 0.01), and the HRs for RFS were 2.08 for N1 (CHA) (*p* = 0.03) and 0.35 for N0 (*p* < 0.01).

Univariable Cox proportional hazards analysis revealed the following prognostic factors for cancer‐specific death (Table 3): female sex (HR 1.38, *p* = 0.05), elevated CA19‐9 levels (≥ 37 U/mL; HR 1.84, *p* < 0.01), poorly differentiated tumor (HR 2.52, *p* < 0.01), advanced T stage (3/4; HR 1.89, *p* < 0.01), microscopic lymphatic invasion (HR 1.93, *p* < 0.01), microscopic venous invasion (HR 2.94, *p* < 0.01), microscopic perineural invasion (HR 3.05, *p* < 0.01), N1 (other) [HR 2.42, p < 0.01; with N0 as the reference], N1 (CHA) [HR 5.45, *p* < 0.01; with N0 as the reference], R1 resection (HR 2.44, *p* < 0.01), and adjuvant chemotherapy (HR 1.41, p < 0.01). In multivariable analysis, the independent prognostic factors were identified as follows: female sex (HR 1.53, *p* = 0.01), elevated CA19‐9 levels (≥ 37 U/mL; HR 1.66, *p* < 0.01), poorly differentiated tumor (HR 2.93, p < 0.01), microscopic venous invasion (HR 1.86, *p* = 0.02), N1 (other) (HR 1.86, *p* < 0.01), N1 (CHA) (HR 3.34, *p* < 0.01), and R1 resection (HR 2.26, p < 0.01).

**TABLE 3:** Univariable and multivariable Cox proportional hazard model analysis of factors affecting CSS.

Variables		*n*	Univariable			Multivariable		
			HR	95% CI	*p*	HR	95% CI	*p*
Age, years	< 75	200						
	≥ 75	91	0.89	0.63–1.25	0.50			
Sex	Male	209						
	Female	82	1.38	1.00–1.90	0.05	1.53	1.10–2.13	0.01
Total‐bilirubin, mg/dL	< 5.0	205						
	≥ 5.0	86	1.25	0.91–1.72	0.17			
CA19‐9, U/mL	< 37	134						
	≥ 37	157	1.84	1.35–2.52	< 0.01	1.66	1.20–2.30	< 0.01
Tumor diameter, cm	< 5.0	206						
	≥ 5.0	85	1.18	0.85–1.64	0.31			
Tumor differentiation	Others	258						
	Por	33	2.52	1.65–3.83	< 0.01	2.93	1.90–4.53	< 0.01
T‐stage^a^	1, 2	165						
	3, 4	126	1.89	1.40–2.56	< 0.01	1.37	0.99–1.88	0.06
Microscopic lymphatic invasion	No	140						
	Yes	150	1.93	1.42–2.63	< 0.01	1.17	0.83–1.66	0.37
Microscopic venous invasion	No	62						
	Yes	228	2.94	1.86–4.64	< 0.01	1.86	1.13–3.07	0.02
Microscopic perineural invasion	No	52						
	Yes	238	3.05	1.84–5.04	< 0.01	1.38	0.76–2.49	0.29
Lymph node metastasis	N0	155						
(N0 as the reference)	N1 (other)	103	2.42	1.74–3.38	< 0.01	1.86	1.31–2.64	< 0.01
	N1 (CHA)	33	5.45	3.51–8.48	< 0.01	3.34	2.09–5.32	< 0.01
Surgical margin	R0	188						
	R1	103	2.45	1.81–3.32	< 0.01	2.26	1.65–3.10	< 0.01
Adjuvant treatment	Yes	54						
	No	237	0.57	0.40–0.80	< 0.01	0.82	0.57–1.17	0.27

Abbreviations: CA19‐9 carbohydrate antigen 19‐9; CHA, common hepatic artery; CI, confidence interval; CSS, cancer‐specific survival; HR, hazard ratio.

^a^ The T‐factor was determined based on the Union for International Cancer Control (UICC) 8th edition.

### 3.4 Diagnostic Accuracy of N1 (CHA)

**FIGURE 4:**
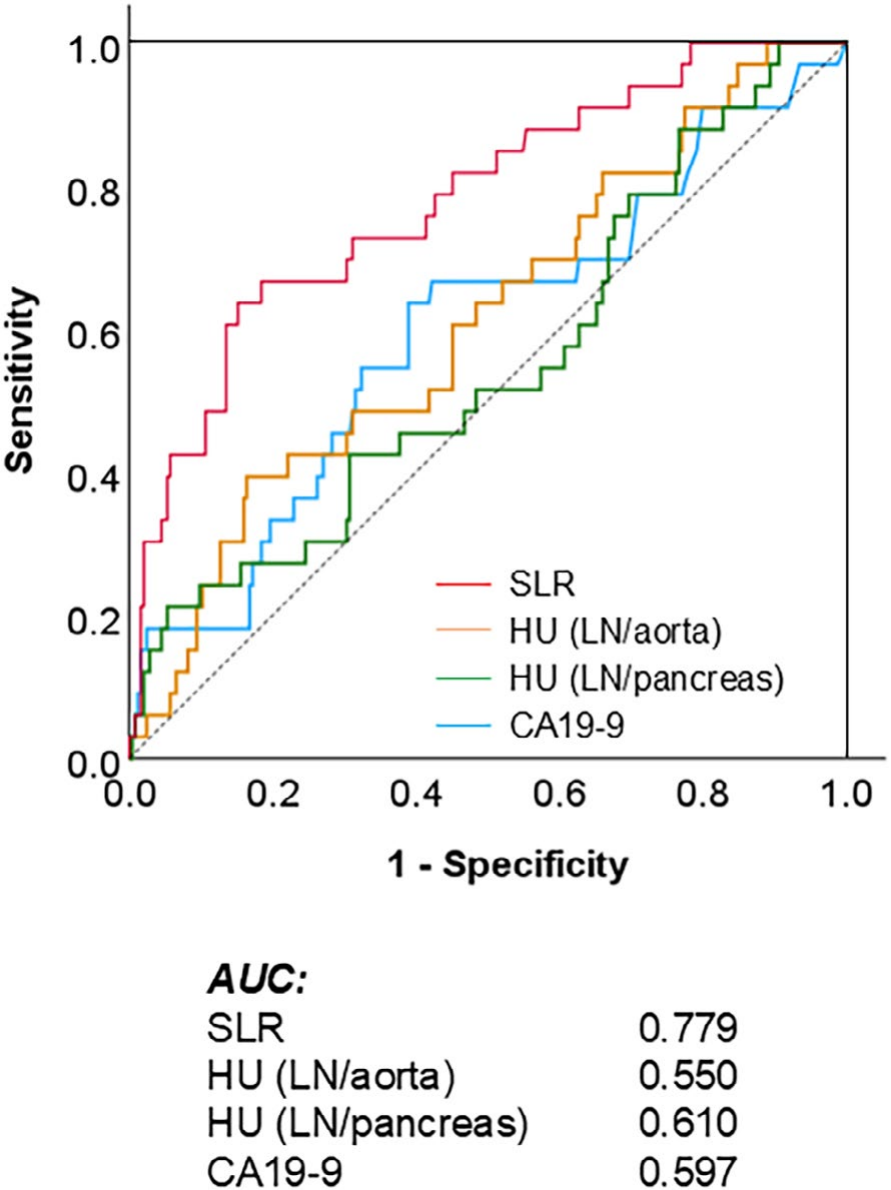
Receiver operating characteristic (ROC) curves of four parameters for predicting N1 (CHA) status. The *y*‐axis represents sensitivity, and the *x*‐axis represents 1–specificity. The area under the curve (AUC) for each parameter is annotated. CA19‐9, carbohydrate antigen 19‐9; HU, Hounsfield unit; LN, lymph node; SLR, short‐to‐long axis ratio.

Figure 4 shows the ROC curves for predicting N (CHA) using the four dependent variables. The AUC of SLR was the highest at 0.779, followed by HU (tumor/pancreas) at 0.610, CA19‐9 at 0.597, and HU (tumor/aorta) at 0.550. The point at which sensitivity and specificity were maximized (Youden index) was 0.57. Among the 62 patients with an SLR of ≥ 0.57, 21 had N1 (CHA) (sensitivity: 63.6%). Among the 216 patients with an SLR < 0.57, 12 had N1 (CHA) (specificity: 94.4%). A total of Thirteen patients could not be evaluated for reasons such as contrast agent allergy or chronic kidney disease.

## 4 Discussion

As multimodal therapy has become increasingly prominent in biliary tract cancer treatment, establishing biological resectability criteria is essential for improving postoperative survival. We demonstrated that patients with N1 (CHA) had an extremely poor 5‐year survival rate (6.9%). Although the N1 (CHA) status was associated with other adverse pathological features, it remained an independent poor prognostic factor in the multivariable analysis. Previous studies have suggested that N (CHA) may have different prognostic impacts in PhCC and DCC, but our study demonstrated that it was a significant adverse prognostic factor in both. Therefore, it was considered reasonable to discuss the findings under the unified category of extrahepatic cholangiocarcinoma. Using the dCECT arterial phase, a higher SLR was significantly associated with lymph node metastasis (sensitivity, 63.6%; specificity, 83.3%). This suggests that the N (CHA) status may be a valuable factor in determining eCCA biological resectability.

Studies have investigated the relationship between lymph node metastasis and prognosis, with increasing attention given to the number of metastatic lymph nodes [15, 16]. According to the latest UICC guidelines, prognostic stratification based on lymph node metastasis is determined using the number of metastatic nodes [7]. Additional indicators such as lymph node ratio and log odds of metastatic lymph nodes have been proposed for PhCC and DCC [17, 18, 19]. In contrast to this numerical approach, we focused on the significance of lymph node metastasis location, specifically N (CHA), and demonstrated its impact on survival outcomes. Although N (CHA) is already recognized as a poor prognostic factor in DCC, this study is the first to demonstrate that it is also a poor prognostic factor in PhCC. Taken together, this is the first report to show the prognostic significance of N (CHA) in extrahepatic cholangiocarcinoma as a whole. As opposed to other regional nodes, this lymph node has a specific size, even under normal conditions, making it easily identifiable on radiographic imaging and has the potential for preoperative diagnosis. However, preoperative determination of the number of metastatic lymph nodes remains highly challenging. Moreover, N (CHA) is readily detectable intraoperatively and accessible early during surgical procedures (see Figure S1), making it a special advantage for early assessment during surgery [20].

Tumor staging is recommended using various imaging modalities, including dCECT, magnetic resonance imaging, and, in certain cases, positron emission tomography [21]. However, the accuracy of lymph node metastasis detection via imaging remains low, with a sensitivity and specificity of 40%–50% and 77%–92%, respectively [22]. This is owing to the difficulty in distinguishing metastatic lymph node enlargement from inflammation‐induced lymphadenopathy, such as that caused by cholangitis [23]. Reports on radiomics, a novel analytical method, have recently increased, and it can become a standard method in the future; however, at present, it has not been established [24]. In this study, we attempted prediction using parameters such as SLR (reflecting roundness), HU value (reflecting contrast enhancement), and CA19‐9 levels (a traditional biological factor of biliary tract cancer). Among these, SLR yielded the most reliable predictive results. Although the sensitivity was 63.6%, the specificity was as high as 94.4%, indicating that lymph nodes with a large SLR are highly likely to be metastatic. Furthermore, even when attempting to construct predictive models by combining SLR with other parameters, none of the individual parameters demonstrated sufficient sensitivity on their own. As a result, no combination surpassed the sensitivity achieved by SLR alone (details of the analysis are omitted for brevity). Although the SLR utility is debatable, a previous study revealed its excellent performance in predicting lymph node metastasis in gastrointestinal cancer [25]. The parameters in our study had an AUC < 0.8, indicating that none offered definitive predictive accuracy. Staging laparoscopy and preoperative EUS‐guided lymph node biopsy are currently the most reliable methods for evaluating lymph node metastasis [26].

Chemotherapy for biliary tract cancer has been standardized for over a decade using a gemcitabine plus cisplatin regimen [27]. However, recent advancements, including the accumulation of new evidence, the emergence of immune checkpoint inhibitors, and molecular‐targeted therapies based on genetic mutations, have rapidly improved treatment outcomes and diversified therapeutic options [28, 29]. Consequently, reports on conversion surgery have begun to emerge, and treatment outcomes continue to improve [30]. Identifying anatomical and biological resectability is a significant clinical challenge in establishing optimal multimodal treatment strategies. When the 5‐year CSS and RFS rates were compared, the differences were +0.3%, +4.9%, and +9.8% in the N (CHA), N (other), and N0 groups, respectively, showing a trend of increasing discrepancy as nodal status improved (Figure 2). This indicates that recurrence was more directly linked to cancer‐related death in the N (CHA) group than in the N (other) and N0 groups, suggesting that aggressive multimodal treatment aimed at suppressing recurrence may be particularly beneficial for patients with N (CHA). Additionally, N (CHA) is a promising candidate for imaging‐based diagnosis, can be targeted using EUS‐guided procedures, and is a suitable staging laparoscopy target. Given that extended lymphadenectomy has been refuted by randomized control trials in cholangiocarcinoma and that the appropriate extent of lymphadenectomy has not been established, preoperative chemotherapy may offer a potential solution to overcome this challenge [31].

This study had some limitations. First, it was a retrospective observational study that introduced a potential selection bias in treatment strategies. Additionally, dCECT data were missing in some cases owing to impaired renal function or contrast agent allergies. Furthermore, the study period spanned 20 years, during which minor changes in treatment strategies occurred. However, the surgical approach to lymphadenectomy remained consistent. Although the dCECT quality changed, the SLR was relatively unaffected. Second, this was a single‐center study with a relatively small sample size. To enhance the statistical power, conducting a multicenter study with prospective observations is essential. Third, the incidence of station 14 metastasis was low (2 cases), and only 13% of patients had metastases involving two or more stations, which is lower than previously reported. At our institution, lymph nodes near the tumor are not separated from the specimen before fixation, which may limit the accuracy of station‐specific evaluation. However, N (CHA), the main focus of this study, could be reliably identified due to its anatomical distance from the bile duct and tumor. The median number of dissected lymph nodes was 11 in PhCC and 19 in DCC, which is sufficient for accurate nodal staging and comparable to previous reports. Finally, the measured parameters were subjectively assessed by the research team. Therefore, independent validation using blinded evaluators and external cohorts is necessary to confirm their clinical utility.

## 5 Conclusions

Lymph node metastasis around the CHA may serve as a prognostic biomarker for poor outcomes in PhCC and DCC. Given that lymph nodes around the CHA are easily identified on preoperative imaging and accessed during surgery, they may serve as crucial determinants in patient stratification for multimodal treatment strategies.

## Conflicts of Interest

The authors declare no conflicts of interest.

## Supporting information


**Figure S1:** Intraoperative identification of N (CHA) during laparoscopic surgery. This lymph node is easily visualized and accessible in the early phase of abdominal surgery.

**Figure S2A:** Cancer specific survival (CSS) and recurrence free survival (RFS) curves in patients with perihilar cholangiocarcinoma (PhCC).

**Figure S2B:** Cancer specific survival (CSS) and recurrence free survival (RFS) curves in patients with distal cholangiocarcinoma (DCC).

## Data Availability

The data that support the findings of this study are available on request from the corresponding author. The data are not publicly available due to privacy or ethical restrictions.
