# Supplementary figures and images for: Lymph Node Metastasis Around the Common Hepatic Artery Is Associated With Dismal Prognosis in Patients Undergoing Resection of Extrahepatic Cholangiocarcinoma
Source: J Hepatobiliary Pancreat Sci. 2025 Jul 29;32(10):756–65. doi: 10.1002/jhbp.12194 (PMC12559879; doi:10.1002/jhbp.12194)

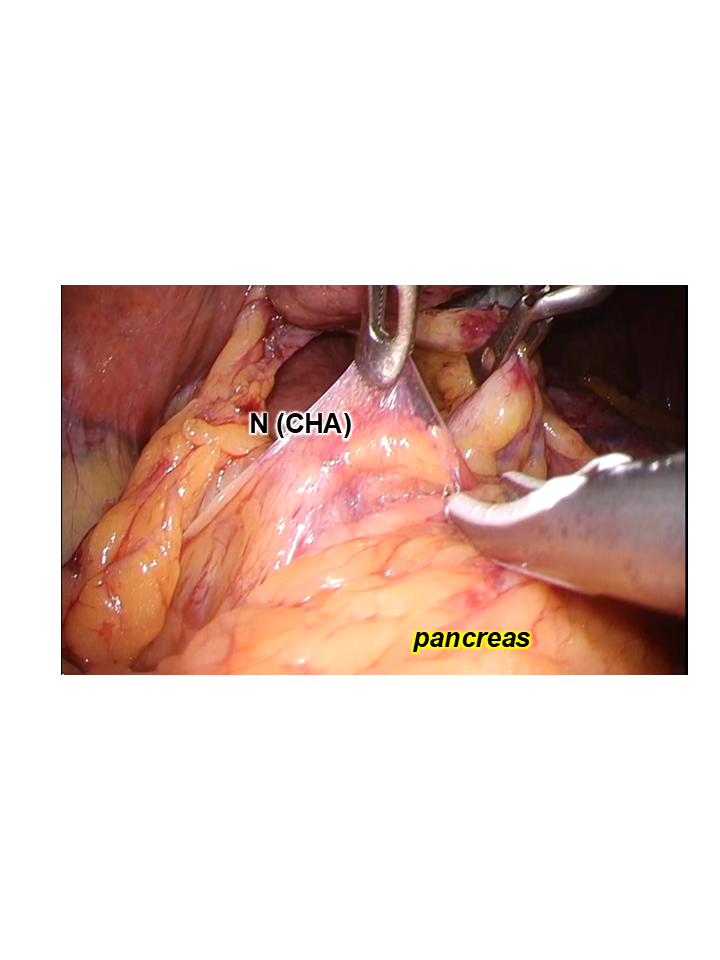

Supplement: Supplementary file 1 — Figure S1: Intraoperative identification of N (CHA) during laparoscopic surgery. This lymph node is easily visualized and accessible in the early phase of abdominal surgery. [file JHBP-32-756-s001.tif]

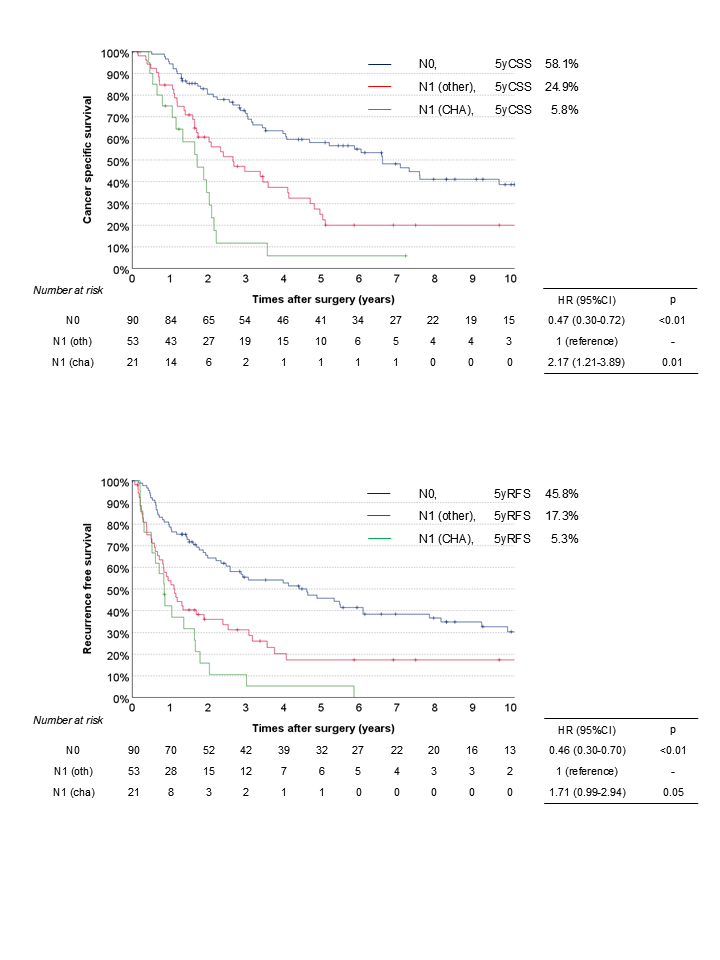

Supplement: Supplementary file 2 — Figure S2A: Cancer specific survival (CSS) and recurrence free survival (RFS) curves in patients with perihilar cholangiocarcinoma (PhCC). [file JHBP-32-756-s003.tif]

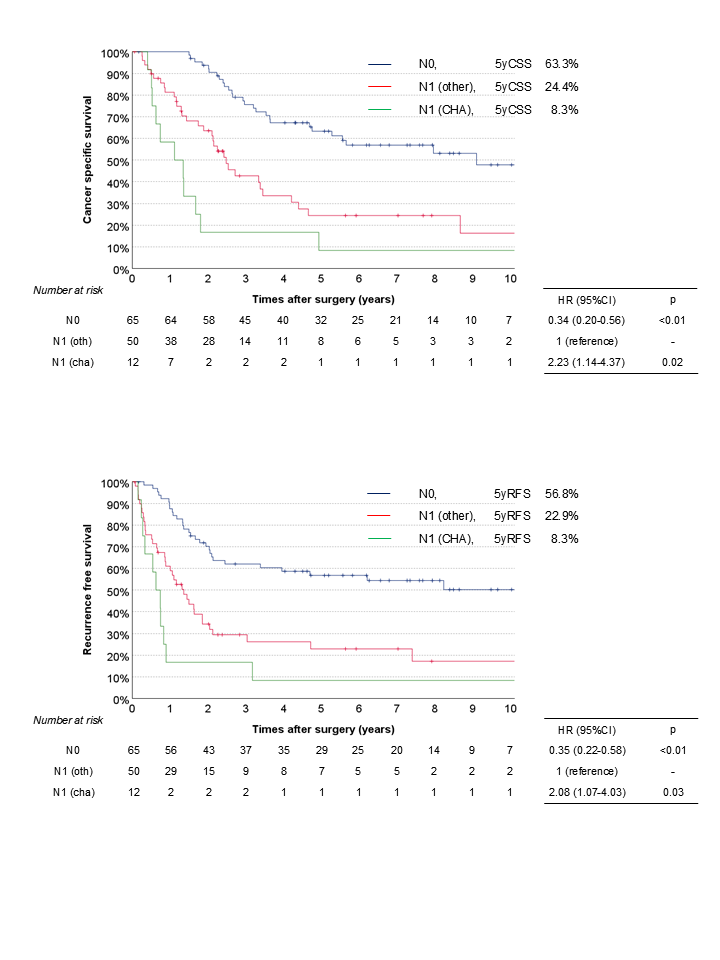

Supplement: Supplementary file 3 — Figure S2B: Cancer specific survival (CSS) and recurrence free survival (RFS) curves in patients with distal cholangiocarcinoma (DCC). [file JHBP-32-756-s002.tif]
